# Association Between Gastrointestinal and Sleep Problems in the General Population of Japan: A Cross-Sectional Community-Based Observational Study

**DOI:** 10.7759/cureus.65311

**Published:** 2024-07-24

**Authors:** Tetsuya Akaishi

**Affiliations:** 1 Department of Education and Support for Regional Medicine, Tohoku University Hospital, Sendai, JPN

**Keywords:** sleep quality, sleep disturbances, relationship, general population, gastrointestinal disorders

## Abstract

Background and aim

Poor sleep is known to be associated with functional gastrointestinal (GI) problems in the general population, but the exact mechanisms underlying the relationship remain unclear. Deeper insights into the exact mechanisms underlying the connection may benefit individuals suffering from these conditions without efficient treatments. Therefore, this study investigated the association between functional GI symptom levels and sleep-related characteristics in the general population of Japan.

Methods

In this cross-sectional questionnaire-based observational study, data including the self-reported level of poor sleep and functional GI symptoms in the last one month were collected from consecutive individuals who visited a hospital in Miyagi Prefecture, Japan, for regular medical checkups between April 2020 and March 2023. The levels of other physical and mental conditions, such as stress at home, fatigability, irritability, thermoregulatory problems, and edema in the limbs, were measured with an 11-point Numerical Rating Scale (NRS) between 0 and 10. Additional sleep-related specific information, such as sleep length, wake-up time, bedtime, trouble falling asleep, and nocturnal awakening, were further collected. Correlations with functional GI symptoms in these characteristics were evaluated by bivariate correlation analyses and generalized regression analyses.

Results

A total of 151 consecutive adults aged ≥18 years (77 males and 74 females) participated in this study. In bivariate correlation analyses, chronic GI symptom levels were significantly correlated with stress at home (p=0.0005), fatigability (p=0.0008), irritability (p=0.0022), edema in the limbs (p<0.0001), and sleep problem (p<0.0001). In the following generalized regression analysis, significant correlations with GI symptom levels were observed in sleep problems (p=0.0042) and edema in the limbs (p=0.0256). Further bivariate correlation analyses using sleep-related subscales revealed that trouble falling asleep in bed (p=0.0001), midnight awakening (p=0.0143), and wakeup time (p=0.0465) were correlated with GI symptom levels. In the following generalized regression analysis, a significant correlation with GI symptom levels was observed in trouble falling asleep (p=0.0239).

Conclusion

Functional GI symptoms in the general population of Japan were associated with poor sleep, even after adjusting for daily stress and irritability levels. Among the sleep-related characteristics, trouble falling asleep was associated with GI dysfunctions. Assessing sleep-related information and achieving good sleep quality with smooth sleep induction may be a clue to relieving GI dysfunctions with uncertain causes.

## Introduction

Sleep problems are known to underlie a wide spectrum of physical and mental health problems in the general population. One such disease spectra linked with sleep disturbance is gastrointestinal (GI) symptoms, including functional GI diseases like functional dyspepsia and irritable bowel syndrome [1-3].

Each prevalence of functional GI diseases and sleep disturbances among the general population is thought to be higher than 10% [4-8], and establishing the mechanisms linking these conditions and efficient therapeutic interventions against the connection is of urgent need in public health. The exact mechanisms linking sleep problems and digestive symptoms still remain uncertain. An issue that makes the evaluation difficult is the potential confounding effect of the mental health conditions (e.g., daily stress at home and irritability) and sleep apnea, which must be adjusted for before deciding the direct linkage between them [9,10].

A previous community-based cross-sectional surveillance from the US demonstrated that the linkage between sleep problems and GI symptoms was independent of obesity levels measured by body mass index [11]. Meanwhile, another one-year follow-up study enrolling patients with functional dyspepsia from China revealed that sleep problems at baseline and dyspeptic symptom scores at one-year follow-up were not longitudinally correlated [12]. The absence of a significant correlation in the longitudinal analysis suggests the presence of longitudinal fluctuations in the symptoms and difficulty in elucidating the causality between them. Moreover, which of the specific sleep-related characteristics, such as sleep length, trouble falling asleep, and midnight awakening, are the most associated with GI symptoms currently remains unknown. In trying to estimate the direct causality between poor sleep and functional GI symptoms, correlation analyses and multivariable regression analyses including such detailed sleep-related information are essential.

To elucidate the link between poor sleep and GI symptoms independent of mental distress, the present study comprehensively collected data regarding the recent levels of GI symptoms, sleep problems, stress, and irritability, from individuals in the general population of Japan. Furthermore, to elucidate the specific sleep-related characteristics being associated with GI symptoms, the correlations between detailed sleep-related subscales and GI symptoms were further investigated.

## Materials and methods

Overview

This is a community-based cross-sectional observational study using self-reported questionnaires regarding health-related backgrounds and current conditions, designed to investigate the link between sleep and functional GI tract problems after adjusting for mental distress levels in the general population.

Participants

In this study, individuals who visited the Kesennuma City Municipal Motoyoshi Hospital (Kesennuma, Japan) for annual medical checkups between April 2020 and March 2023 were initially recruited [13]. Those who agreed to participate in the present study and returned the responded questionnaire sheet were enrolled for the subsequent statistical analyses.

Materials

To measure the recent level of each physical and mental condition in the last one month, an 11-point Numerical Rating Scale (NRS) between 0 and 10 was utilized, with 0 representing “no symptom” and 10 representing “the worst level imaginable” [14]. A Face Rating Scale was added to each 0-10 NRS scale bar, with the faces of “no problem” at 0, “little bit” at 2, “little more” at 4, “even more” at 6, “whole lot” at 8, and “worst” at 10.

Procedure

All individuals who visited the hospital for the annual medical checkup were initially recruited to take part in the surveillance. Those who agreed to take part in the project were handed the questionnaire sheets on the spot. Most of the participants answered the questionnaires on the same day of recruitment before leaving the hospital, but some of them took home the sheets and later brought the answered sheets with their written informed consent. Collected data from individuals who did not agree to the use of data for research purposes were excluded from the subsequent analyses.

Variables

From the participants, the age, sex, and alcohol consumption amount were collected as the demographic background. As for the recent physical and mental conditions, levels of the following conditions in the last one month were collected using the 11-point NRS scores: functional GI symptoms (constipation, diarrhea, dyspepsia, and anorexia), stress at home, fatigability, thermoregulatory problem, irritability, edema in the four limbs, and sleep problem. Additionally, the following sleep-related subscales were further collected: weekly average of sleep length, wakeup time in the morning, bedtime at night, prolonged midnight awakening frequency, time needed to fall asleep in bed at night, and monthly average of afternoon sleep length.

Statistical analysis

Distributions of the quantitative variables were described with the median and interquartile range (IQR; 25-75 percentiles). The sample size for the multiple regression was estimated based on the following parameter settings: p-value (α)=0.05, statistical power (1−β)=0.80, expected effect size (f^2^)=0.15, and the number of predictor variables=5, which suggested the minimum required sample size of approximately 90-100 individuals. Bivariate correlation analyses were performed by calculating Pearson’s correlation coefficient (R). The subsequent multivariable linear regression analyses were performed by simultaneously entering the explanatory variables with p-values <0.10 in the bivariate analyses. When the non-normality in the residual of a multiple linear regression model was suggested by the Shapiro-Wilk test, an additional fitting of the generalized linear regression model using the negative binomial maximum likelihood method was further performed. P-values less than 0.05 were considered statistically significant. The p-value threshold was not adjusted for the multiple comparisons based on the exploratory nature of this study. Statistical analyses in this study were performed using the R Statistical Software version 4.1.3 (R Foundation, Vienna, Austria) and JMP Pro version 17 (SAS Institute, Cary, USA).

Ethical considerations

This study was approved by the Institutional Review Board of Tohoku University Graduate School of Medicine in April 2020 (approval number: 2020-1063). Written informed consent was obtained from all of the enrolled individuals. All of the study process was performed in accordance with the latest version of the Declaration of Helsinki, as revised in 2013.

## Results

Population background

A total of 151 consecutive adults aged ≥18 years (77 males and 74 females) who visited the hospital for annual medical checkups were enrolled. The demographic backgrounds of the participants are summarized in Table 1. The median of the age was 43 years (IQR, 31-57 years). The median of the NRS for GI symptom levels in the last one month was 3.0 (IQR, 1.0-5.0), and the same for sleep problems was 3.0 (IQR, 2.0-6.0).

**Table 1:** Demographic data of the enrolled 151 adults * Median and interquartile range GI, gastrointestinal; NRS, Numerical Rating Scale

Characteristics	Frequency/distribution
Male, n (%)	77 (51%)
Younger adults aged 18–64 years, n (%)	132 (87%)
Diabetes mellitus, n (%)	8 (5.3%)
Metabolic syndrome, n (%)	10 (6.6%)
Age *	43 years (31-57 years)
GI tract symptom level with NRS *	3.0 (1.0-5.0)
Sleep problem level with NRS *	3.0 (2.0-6.0)
Stress at home with NRS *	3.0 (1.0-5.0)
Fatigability with NRS *	5.0 (3.0-6.0)
Thermoregulatory problem with NRS *	0.0 (0.0-2.0)
Irritability with NRS *	5.0 (3.0-7.0)
Edema in the limbs with NRS *	2.0 (0.0-4.0)
Daily sleep length (weekly average) *	6.5 hours (6.0-7.0 hours)
Wakeup time (weekly average) *	06:00 AM (05:40 AM-06:55 AM)
Bedtime (weekly average) *	11:00 PM (10:30 PM-12:00 PM)
Prolonged midnight awakening *	0 night/month (0-3 nights/month)
Time needed to fall asleep in bed *	30 minutes (10-35 minutes)
Afternoon sleep length *	150 minutes/month (0-375 minutes/month)

Correlation with the GI symptom level

The calculated Pearson’s R with the GI symptom level for each of the other evaluated characteristics is listed in Table 2. Significant correlations were observed in the level of stress at home (R=0.283, p=0.0005), fatigability (R=0.276, p=0.0008), irritability (R=0.252, p=0.0022), edema in the limbs (R=0.319, p<0.0001), and sleep problem (R=0.366, p<0.0001). Next, the explanatory variables with p-values <0.10 in the bivariate correlation analyses were simultaneously entered into a multiple linear regression model. The result is summarized in the right half of Table 2. As a result, the level of sleep problems (β=0.2642, p=0.0028) and edema in the limbs (β=0.2204, p=0.0078) were suggested to be the significant characteristics in relation to GI symptom levels. However, the obtained residuals were non-normally distributed based (p=0.0012, Shapiro-Wilk test), and an additional generalized linear regression analysis was further performed (Table 3). Again, the level of sleep problems (p=0.0042) and edema in the limbs (p=0.0256) were significantly associated with GI symptom levels.

**Table 2:** Correlations with the GI symptom level for each health-related characteristic In each bivariate analysis, the Pearson’s correlation coefficient (R) with the GI symptom level was calculated. In the multiple linear regression analysis, the sleep-related subscales with p-values <0.10 in the bivariate analyses were used as explanatory variables and the GI symptom level in the last one month was used as the outcome variable. * Dummy variable; “Yes” =1, “No” =0. † Levels in the last one month, reported using the NRS from 0 to 10. GI, gastrointestinal; NRS, Numerical Rating Scale

Characteristics	Bivariate analyses		Multiple linear regression model		
	Pearson’s *R*	P	Standardized *β*	t	P
Age	-0.0611	0.4640	-	-	-
Male *	-0.0437	0.6008	-	-	-
Diabetes mellitus *	-0.0049	0.9532	-	-	-
Metabolic syndrome *	0.0691	0.4073	-	-	-
Stress at home^ †^	0.2830	0.0005	0.0984	1.11	0.2671
Fatigability^ †^	0.2756	0.0008	0.0851	0.94	0.3487
Thermoregulatory problem^ †^	0.1142	0.1700	-	-	-
Irritability^ †^	0.2515	0.0022	-0.0273	-0.29	0.7738
Edema in the limbs^ †^	0.3188	<0.0001	0.2204	2.70	0.0078
Sleep problem^ †^	0.3662	<0.0001	0.2642	3.04	0.0028
Alcohol consumption amount	0.0256	0.7652	-	-	-

**Table 3:** Generalized linear regression for GI symptom level with other condition levels The result was obtained using a generalized linear regression model based on the negative binomial maximum likelihood method. * Levels in the last one month, reported using the NRS from 0 to 10. GI, gastrointestinal; NRS, Numerical Rating Scale

	B	Std. Error	Wald 95% CI		Hypothesis test	
			Lower	Upper	Wald Chi-square	P
Stress at home *	0.0332	0.0301	-0.0257	0.0922	1.2204	0.2693
Fatigability *	0.0288	0.0366	-0.0430	0.1006	0.6178	0.4319
Irritability *	-0.0137	0.0375	-0.0873	0.0599	0.1335	0.7148
Edema in the limbs *	0.0675	0.0302	0.0082	0.1267	4.9853	0.0256
Sleep problem *	0.0771	0.0269	0.0243	0.1298	8.2065	0.0042

Correlation between GI symptom and sleep-related subscales

Based on the finding that the level of GI symptoms and sleep problems in the last one month were closely related to each other, further analyses using sleep-related subscales in view of the association with GI symptoms were performed. The results of the bivariate correlation analyses are listed in the left half of Table 4. Significant correlations were observed in the midnight awakening frequency (R=0.204, p=0.0143) and time needed to fall asleep in bed (R=0.315, p=0.0001). The result of a multiple linear regression analysis using the characteristics with p-values <0.10 in the bivariate analyses is shown in the right half of Table 4. This time, only the time needed to fall asleep in bed at night was significantly correlated with the GI symptom level (β=0.2384, p=0.0076). However, the obtained residuals were non-normally distributed based (p=0.0047, Shapiro-Wilk test), and an additional generalized linear regression analysis was further performed (Table 5). Again, only the time needed to fall asleep was significantly associated with the GI symptom level (p=0.0239).

**Table 4:** Correlations between GI symptom level with sleep-related subscales In each bivariate analysis, the Pearson’s correlation coefficient (R) with the GI symptom level was calculated. In the multiple linear regression analysis, the sleep-related subscales with p-values <0.10 in the bivariate analyses were used as the explanatory variables, and the GI symptom level was used as the outcome variable. GI, gastrointestinal

Characteristics	Bivariate analyses		Multiple linear regression model		
	Pearson’s *R*	P	Standardized *β*	t	P
Weekly sleep length	−0.1394	0.0933	−0.0632	−0.73	0.4641
Wakeup time	0.1656	0.0465	0.1134	1.36	0.1756
Bedtime at night	0.0890	0.3200	–	–	–
Prolonged midnight awakening	0.2038	0.0143	0.1321	1.51	0.1324
Time needed to fall asleep	0.3148	0.0001	0.2384	2.71	0.0076
Afternoon sleep length	0.0646	0.4502	–	–	–

**Table 5:** Generalized linear regression for the GI symptom level with sleep-related specific data The result was obtained using a generalized linear regression model based on the negative binomial maximum likelihood method. GI, gastrointestinal

	B	Std. Error	Wald 95% CI		Hypothesis test	
			Lower	Upper	Wald Chi-square	P
Weekly sleep length	-0.0327	0.0733	-0.1764	0.1110	0.1990	0.6556
Wakeup time	0.0743	0.0691	-0.0613	0.2098	1.1535	0.2828
Prolonged midnight awakening	0.0157	0.0125	-0.0087	0.0402	1.5931	0.2069
Time needed to fall asleep	0.0061	0.0027	0.0008	0.0114	5.1005	0.0239

**Figure 1:**
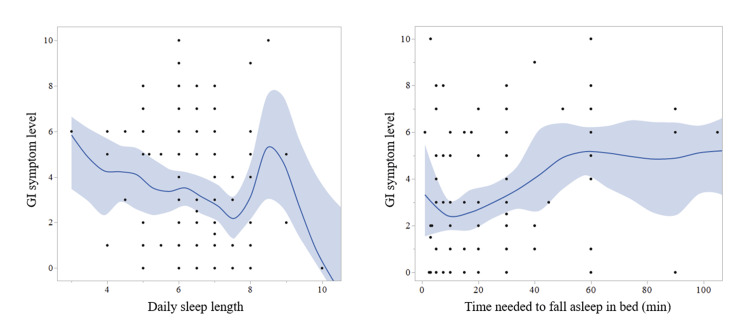
Scatterplots between GI symptom level and sleep-related subscales The time needed to fall asleep in bed at night (right panel), rather than the sleep length (left panel), was suggested to be a sleep-related subscale significantly correlating with GI symptom level. The fitting curves (blue curves) and bootstrap confidence regions for the fit (filled areas) were obtained with curve fittings using piecewise cubic smoothing spline interpolation. GI, gastrointestinal

Based on these findings, a scatterplot with the time needed to fall asleep in bed (X-axis) and GI symptom level in the last one month (Y-axis) was built as shown in the right panel of Figure 1. A clear positive correlation between these two variables could be visually confirmed with the scatterplot, with the lowest GI symptom level among those with 0-20 minutes to fall asleep in bed at night.

## Discussion

In this cross-sectional observational study, a close association between sleep and functional GI symptoms was identified in the general population of Japan. Among the evaluated sleep-related subscales, difficulty in falling asleep was significantly associated with the GI symptom level, whilst the sleep length was not. From before, the potential relationship between sleep quality and GI symptoms has been proposed [11,15]. A cross-sectional community-based study from the Republic of Korea demonstrated significant associations between sleep disturbances and miscellaneous GI symptoms including acid regurgitation and abdominal distension [1]. Another meta-analysis revealed that sleep disorder is associated with an elevated prevalence of irritable bowel syndrome [16]. Most of the other previous cross-sectional studies also agreed on the presence of a correlation between poor sleep and GI symptoms. The present study also agreed on the correlation between these two conditions. Moreover, this study demonstrated that the linkage between poor sleep and functional GI symptoms was still significant even after adjusting for the daily stress and irritability levels. Direct pathophysiological mechanisms linking these two conditions independent of mental conditions were suggested. Although the causality between these two is uncertain, the findings may indicate the benefit of assessing and treating the sleep quality in each individual who suffers from chronic GI tract dysfunction with uncertain causes.

The exact mechanisms linking sleep problems and GI symptoms still remain uncertain. Sleep dysfunction is reported to result in an alteration in serum proinflammatory cytokines and chemokines, which may cause miscellaneous GI tract disorders like gastroesophageal reflux disease, inflammatory bowel disease, and liver disorders [17]. These GI symptoms may further deteriorate sleep disturbances, possibly creating an interplay between sleep problems and GI symptoms. Other proposed potential mechanisms underlying GI symptoms among those with sleep problems included disturbed hypocretin/orexin signaling, melatonin dysregulation, and gut microbiota alterations [18-20]. It has been experimentally demonstrated that microbiome diversity is positively correlated with sleep efficiency and sleep length, whilst it was negatively correlated with sleep fragmentation [21]. Another previous study indicated that subjective perception of sleep quality in each individual may be the most influential and predictive for subsequent GI symptom levels [22].

Because of the cross-sectional nature of this study, it could not determine the causal relationship between sleep and GI symptoms. It may be reasonable to think that good sleep quality contributes to realizing better GI tract function. There is an interventional study demonstrating that achieving better sleep quality by administering sleep aids will bring improvement in GI symptoms in those with functional dyspepsia [23]. Another recent study employing the Mendelian randomization analysis revealed that insomnia may induce irritable bowel syndrome, but not vice versa [24]. Meanwhile, an influence in the opposite direction from the GI tract environment to sleep quality may be also conceivable [21]. For example, several previous studies indicated that including probiotics in the diet may improve sleep quality, although heterogeneity of data in the literature was suggested [25,26]. Or, there is a possibility that reflux symptoms when lying in bed among those with dyspeptic predisposition may disturb the sleep quality [27]. Considering together, a reciprocal causal association may exist between sleep quality and GI symptoms [28]. To conclude the exact causal relationship, longitudinal follow-up or interventional studies in the future are needed.

Another notable finding of this study is that trouble falling asleep is one of the most important sleep-related characteristics associated with functional GI symptoms. This finding is consistent with a previous study showing a linkage between trouble falling asleep and rectal urgency [11]. However, again, the exact mechanisms linking these two conditions or the direction of causality between them remain unknown yet. To conclude the benefit of achieving good sleep quality in relieving functional GI symptoms, interventional studies utilizing hypnotics, chronotherapy, or guidance to avoid sleeping late at night are needed.

There are several limitations of this study. First, this study was with a relatively small sample size. Future studies with larger sample sizes are needed to verify the obtained findings in this study. Another limitation was that this study exclusively enrolled individuals with Asian ancestry, and the generalizability of the obtained findings to other races and ethnicities remains unknown. Further studies from other countries are needed to confirm this. Moreover, because of the cross-sectional nature of the present study, the direction of causality between sleep disturbances and GI tract dysfunctions could not be estimated. Finally, the present study did not utilize sleep-quality assessment tools like the Pittsburgh Sleep Quality Index and Athens Insomnia Scale. Assessment of sleep quality using the NRS alone may not be sufficient due to the lack of its validation. Future studies would be better to use these established assessment tools for sleep quality.

## Conclusions

Functional GI symptoms in the general population of Japan were closely associated with poor sleep, represented by difficulty in falling asleep. The significance of the connection was still observed even after adjusting for daily levels of stress and irritability. Carefully assessing sleep-related information and improving sleep quality to achieve smooth sleep induction may be helpful in relieving functional GI symptoms with uncertain causes. Further studies to elucidate the mechanisms linking sleep problems and functional GI symptoms are warranted.
